# Taping for an Acute and Subacute Patellofemoral Dislocation, Recurrent Subluxation

**DOI:** 10.1177/26350254251346800

**Published:** 2025-10-16

**Authors:** Jenny McConnell

**Affiliations:** † McConnell Physiotherapy Group, Mosman, New South Wales, Australia; ‡ Centre for Exercise and Sports Medicine, University of Melbourne, Parkville, Victoria, Australia; Investigation performed at McConnell Physiotherapy Group, Mosman, Australia

**Keywords:** patellofemoral, dislocation, subluxation, instability, taping

## Abstract

**Background:**

A primary acute patellofemoral dislocation needs to be managed well to ensure adequate tissue healing. Evidence that surgical intervention is better than conservative management is equivocal, particularly in the skeletally immature individual. Conservative management is less expensive and less invasive, and it may be better at maintaining knee function than surgical intervention. Along with appropriate immobilization, using rigid strapping tape, conservative management should consist of a tailored rehabilitation program to improve dynamic lower limb loading and quadriceps control, so individuals may be able to return to their previous active lifestyle, including their sporting activities.

**Indications:**

The initial phase of a primary patellar dislocation, followed by the subacute phase, including recurrent patellar instability.

**Technique Description:**

The first sequence demonstrates how to tape the patella for the first 6 weeks following an acute patellofemoral dislocation. The rigid tape needs to tilt and center the patella in the trochlea, ensuring that the inferior pole does not aggravate the highly nociceptive infrapatellar fat pad. The medial soft tissues are shortened to enhance healing of the elongated tissues. The second sequence demonstrates taping for a subacute dislocation (after 6 weeks) or a recurrent subluxing patella. Firm tape can be applied across the vastus lateralis to minimize lateral patellar displacement, facilitating vastus medialis oblique contraction.

**Results:**

These taping techniques, combined with a guided exercise program focused on specific lower limb training to improve neural patterning, have enabled patients to successfully return to their previous sporting activities. They should still be taped for sports to minimize the incidence of recurrence. The return to sport is gradual, allowing individuals to feel confident. Depending on the amount of contact and pivoting activity in the sport, as well as the severity of the dislocation, it may take 12 months before some are ready to fully return to their sport.

**Discussion/Conclusion:**

With appropriate initial stabilization of the soft tissues, graduated taping in the subacute or recurrent phase, and a tailored exercise program, individuals who have had an acute dislocation can be effectively rehabilitated so that they can return to their previous activity levels.

**Patient Consent Disclosure Statement:**

The author(s) attests that consent has been obtained from any patient(s) appearing in this publication. If the individual may be identifiable, the author(s) has included a statement of release or other written form of approval from the patient(s) with this submission for publication.

## Graphical abstract

**Graphical Abstract:**
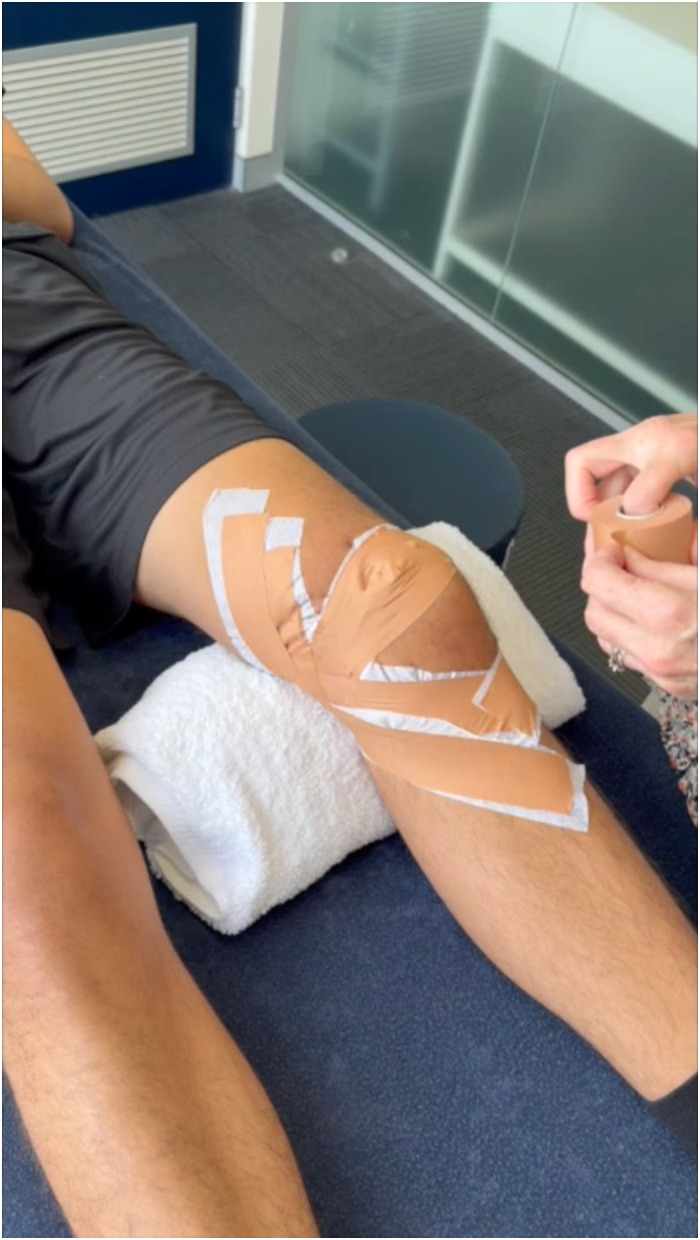
This is a visual representation of the abstract.

## Video Transcript

## Background

Hello, this is Jenny McConnell. I am going to show you how to tape for an acute patellofemoral (PF) dislocation—that is, for the first 4-6 weeks post-dislocation, as well as subacute dislocation and recurrent subluxation.

Evidence that surgical intervention is better than conservative management is equivocal, particularly in the skeletally immature individual.^2,9,11^ Conservative management is less expensive and less invasive, and it may be better at maintaining knee function than surgical intervention.^2^

Along with appropriate immobilization, using rigid strapping tape, conservative management should consist of a tailored rehabilitation program to improve dynamic lower limb loading and quadriceps control, so individuals may be able to return to their previous active lifestyle, including their sporting activities.^5,6,7^

### Taping for Acute PF Dislocation

For the first 4-6 weeks immediately after acute PF dislocation:^5,6^

- Tape is replaced at 3 weeks.
- Tape is applied with the knee flexed to 30°.
- Tape should limit full extension and limit flexion >90°.
- Under-wrap cloth tape is applied to protect skin, followed by firm application of rigid rayon tape.
- The first 2 pieces of rigid tape tilt and glide the patella medially.
- The next 4 pieces of tape are directed at unloading (shortening) the medial soft tissue structures—medial retinaculum, medial PF ligament (MPFL), and vastus medialis oblique (VMO)—and to limit knee flexion.
- The final piece further unloads the infrapatellar fat pad (IFP), which can be impinged during a dislocation.^8^

### Taping for Subacute (6-12 weeks) PF Dislocation and Recurrent Subluxation

Taping for the subacute dislocation is for 6-12 weeks post-dislocation, and this tape can be also be used for the recurrent subluxation:

- Tape is positioned with the patient's knee in a relaxed position, resting on the plinth, so the patella can engage in the trochlea.
- Tape tilts, centers the patella in the trochlea, and rotates the patella,^5,6^ ensuring that the inferior pole does not aggravate the highly nociceptive IFP.^1,3,8,10^
- The medial soft tissues are shortened to enhance healing of the elongated tissues.^6^
- The patella is rotated either clockwise or anticlockwise to further improve the patella's engagement in the trochlea.
- Inhibitory tape is required to minimize the lateral pull of the vastus lateralis (VL) and enhance VMO.^4,12^

### Results

- Specific taping, a guided exercise program based on specific lower limb training, including gluteals, quads, and foot musculature to improve neural patterning and, if necessary, appropriate stretches of tight anterior hip structures, hamstrings, and calf muscles → successful return to sport.^5,8^
- Patients need to be taped for sport to minimize the incidence of recurrence.
- Return to sport is gradual (6-12 months), depending on the amount of contact and pivoting activity in the sport, as well as the severity of the dislocation.
- To maintain optimal PF function, patients need to do the specific neural weightbearing training every day for the rest of their lives (like cleaning their teeth).
- These exercises should take no more than 5 minutes, require no equipment, and can be done anywhere in conjunction with their everyday life.

### Precautions

- Two types of skin problems can arise:
  i) Friction rub, where the skin on the medial side of the knee can blister
    - Too rigorous application or removal of tape can cause friction rub—ease tension off when taping toward hamstrings, and gently ease tape off when removing tape.
    - Additional skin protection can be provided by skin protector wipes or calamine lotion.
    - Generally, this does not occur with acute dislocation as movement of the knee has been limited and the patient is not active.
  ii) Allergic reaction (there is usually a delay of 3-4 weeks before a skin reaction), usually 5% to 10%
    - If skin becomes itchy, use an oral antihistamine medication.
    - Extra skin protection usually mitigates the risk.
    - May have to stop taping.

### Conclusion

Tape is an adjunct to treatment—the patient should be given weightbearing quadriceps and gluteal exercises to improve dynamic lower limb loading and PF stability. Return to sport is gradual as the patient needs to feel confident in PF stability. Tape must be in situ for sporting activities. Individuals who have had an acute dislocation can be effectively rehabilitated so that they can return to their previous activity levels.

## Technique Description

### Taping for an Acute PF Dislocation

The patient needs to be in slight knee flexion, no more than 30°, with a couple of rolled towels under the knee when the knee is being taped. The patient will not be able to straighten the knee anyway because of pain and swelling. The white hypoallergenic tape is placed gently on the skin to minimize friction on the skin from the more rigid brown tape. The aim of the rigid brown tape is to enable healing of the soft tissue structures. The first piece of rigid tape starts in the middle of the patella to tilt the patella over to correct any lateral tilt, while at the same time, the soft tissues on the medial side are lifted gently toward the patella to shorten the tissues. Then I am going to take a slightly longer piece to position the patella in the trochlea. This tape starts just past the lateral border of the patella, while again lifting the soft tissues up gently on the medial side.

I am trying to shorten the soft tissue, so next I am going to really try to shorten the medial structures by really bringing them together. So, I do that distally and proximally. I am also going to unload the fat pad because it is often damaged during a dislocation. With the final 2 pieces of tape I put on, I am going to come much further around the medial side of the knee. I am going to come right around underneath with these tapes, which are designed to minimize knee flexion. So, with these tapes, the aim is to restrict knee flexion to less than 90°.

So, the range of motion in the knee should be limited from 30° to 90°. Lifting up on the medial side really gives the medial soft tissue structures a great deal of support by shortening them, which optimizes healing. I again come proximally, lifting the soft tissues down toward the knee.

### Subacute Patellar Dislocation and Recurrent Patellar Subluxation

Remember, a lot of these patients are described as having patellas that are like wet soap on glass, meaning they're very mobile. The patella goes in all sorts of directions, so we have to encourage the VMO to work well and decrease the amount of activity in the lateralis. At this stage, the patient requires more support on that medial side to enhance healing of the medial structures. The first thing I am going to do is protect the skin. I am going to make sure that the tape is half on and half off, so I am going to feel the inferior pole and superior pole and put my tape over the superior half of the patella, so I do not run the risk of pushing the patella down into the fat pad and aggravating the fat pad. The fat pad is a highly nociceptive structure, so I have to make sure the patella does not ride down into the fat pad, as the patient will experience a lot of pain. I am also going to support the fat pad because often it can be damaged during a dislocation, so I need to shorten the tissues to minimize the risk of further inflammation of the fat pad.

With the first piece of tape, I am going to feel the patella and make sure I am right in the middle, so this piece of tape will tilt the lateral border medially, tilting the inferior pole of the patella up. I am trying to make the patella more parallel with the femur so that when I put my glide tape on, it will relocate the patella into the trochlea, so the patella remains centered. So now I have shortened the tissues, the VMO is going to work better. I am now going to physically rotate the patella. In this case, because it is his left leg, I am going to rotate the patella clockwise. If it were his right leg, I would be rotating the patella anticlockwise. I am going to start in the middle of the patella, and I am lining my tape up with the opposite shoulder. I am going to turn, with my proximal hand, the superior pole of the patella clockwise, and then I am going to rotate the inferior pole around, so the actual tape looks a bit like a back-to-front Nike tick. So now I am going to start on the superior pole, and again I am going to line the tape up with the opposite shoulder. I am rotating the superior pole, again clockwise, while I turn the inferior pole of the patella toward the midline with my distal hand, which is clockwise, so I am making an upside-down Nike tick. I have really controlled the rotation. So, when the patient contracts their quads now, the tape minimizes some of the lateral displacement. To stop the inferior pole from going into the fat pad and aggravating that, I am going to unload the fat pad. I am going to start right at the tibial tubercle, coming wide out to the joint. I am going to lift the soft tissue up very firmly, so it is going to ooze over the top. I am aiming for a muffin top, so I am really wanting that to ooze. I keep it wide so that the person can bend their knee. I am going to come again on the tibial tubercle; this time I am going lateral, very wide out. This tape does not touch the other tape on the knee. The first one touches the tape on the medial side; this one does not. I lift the soft tissue up very firmly and anchor the tape down. So now I have a nice muffin top to take the load off the fat pad, so when the patient tightens their quads, the patella does not actually push down into the fat pad.

So now I am going to show you how to squash the lateralis so that when the patient contracts their quads, the lateralis works less and the medialis is enhanced. We need to ensure the VL does not work as much and encourage the VMO to work a bit better, so what I am going to do is squash the lateralis. I am going to come across the belly and start posterolaterally, putting my finger in firmly to make a divot and really giving the tape a firm pull. You can see a divot through there, and I am going to do another one. The VMO is now working more strongly, and there is less of a lateral pull on that patella.

This is a very good way to help rehabilitation, by improving VMO activation and minimizing lateral subluxation of the patella.

## Supplemental Material

sj-docx-1-vjs-10.1177_26350254251346800 – Supplemental material for Taping for an Acute and Subacute Patellofemoral Dislocation, Recurrent Subluxation Supplemental material, sj-docx-1-vjs-10.1177_26350254251346800 for Taping for an Acute and Subacute Patellofemoral Dislocation, Recurrent Subluxation by Jenny McConnell in Video Journal of Sports Medicine
