# Supplementary material for: Taping for an Acute and Subacute Patellofemoral Dislocation, Recurrent Subluxation
Source: Video J Sports Med. 2025 Oct 16;5(5):26350254251346800. doi: 10.1177/26350254251346800 (PMC12536177; doi:10.1177/26350254251346800)
Supplement: sj-docx-1-vjs-10.1177_26350254251346800 – Supplemental material for Taping for an Acute and Subacute Patellofemoral Dislocation, Recurrent Subluxation [file sj-docx-1-vjs-10.1177_26350254251346800.docx]

**More detailed information on taping for acute patellofemoral dislocation, subacute dislocation or recurrent subluxation and the subsequent rehabilitation**

**Background**

A primary acute patellar dislocation is a common knee injury, accounting for 3% of acute knee injuries, seen in emergency departments, with the incidence being 5.8 per 100,000 people per year. Two-thirds of acute patellar dislocations occur in active young patients (majority between 12-18 years) ^(1,2,3)^. Acute patellar dislocations need to be managed well to ensure adequate tissue healing. Evidence that surgical intervention is better than conservative management is equivocal, particularly in the skeletally immature individual ^(4,5,6)^. Conservative management is less expensive, less invasive and may be better at maintaining knee function than surgical intervention ^(6)^. Along with appropriate immobilisation, using rigid strapping tape ^(7)^, conservative management should consist of a tailored rehabilitation programme to improve dynamic lower limb loading and quadriceps control ^(8,9)^, so individuals may be able to return to their previous active lifestyle, including their sporting activities.

It is essential that the patellofemoral joint can be appropriately immobilised, with tape, as soon as possible after the acute dislocation, to ensure optimum tissue healing. Once the tape is applied, the patient will be able to weight bear immediately through the leg, which will minimise the muscle wasting of the injured limb. Usually, crutches are only required for a few days post dislocation, as the patient feels very secure with the tape in situ. The tape will need to be changed at 3 weeks as the swelling subsides, to maintain adequate protective immobilisation.

**Technique**

The tape is applied with the patient supine and the injured knee supported with a towel, so it is generally flexed to 20-30 degrees. Full extension after an acute dislocation is too painful for the patient to maintain and because of pain and apprehension, the patient will not be able to relax their hamstrings. Also, when there is a large effusion in the knee joint, it is difficult and causes great discomfort for the patient if the knee is not supported in a small degree of flexion. The tape is applied to minimise strain on the MPFL, medial retinaculum and to centre the patella in the trochlea, improving the ability of the vastus medialis oblique (VMO) to contract and provide some protection to the strained/ruptured medial structures by centring the patella in the trochlea to decrease the unchecked pull of the lateral structures. The largest change in strain in the MPFL occurs between 25 and 30 degrees (10% increase), with the highest value of strain occurring at 120 degrees of knee flexion ^(10)^.

After making sure the skin is clean and the leg hair is removed (hair clippers are generally the best option for this), a hypoallergenic woven cloth tape is placed gently on the superior half of the patella to protect the skin. This tape starts just past the lateral border of the patella, coming around the medial border of the knee to the hamstrings tendon, then two pieces of the same tape are placed on the medial side of the knee, the first extending from the tibial tubercle to the medial femoral condyle, the second extending from the medial femoral condyle to middle of the anterior aspect of the thigh, a quarter way of the femoral length from the patella. The corrective tape (non-rigid flesh coloured tape) starts in the middle of the patella to correct a lateral and posterior patellar tilt (tilt tape). It is imperative that the inferior pole of the patella is tilted superiorly to minimise any aggravation of the highly nociceptive infrapatellar fat pat (IFP) ^(11,12,13)^. The tape is firmly positioned across to the medial side of the knee, going posteriorly stopping short of the end of the under-tape. While the rigid tape is being applied, the medial soft tissues are lifted gently towards the patella to minimise skin friction. The second piece of tape corrects a lateral glide (glide tape) and starts just lateral to the patella and is positioned over the first tape ^(14)^. The next two pieces of tape will shorten the medial soft tissues. While they are being applied the soft tissue is being lifted each time towards the patella. Two further pieces are placed proximally and distally to the first medial tapes. These tapes come further posteriorly on the knee, into the popliteal fossa to limit flexion, so the patient’s knee movement is effectively restricted to less than 90 degrees of flexion. The soft tissue is again lifted towards the patella to ensure maximally shortening of the medial soft tissue structures. This taping technique gives a great deal of confidence to the patient about the stability of their knee. Patellar positioning may be further enhanced by rotating the inferior pole of the patella internally and the superior pole externally, which is rotation in a clockwise direction for a left knee and anticlockwise direction for a right knee.

Six weeks after an acute dislocation or if the patient has a chronically subluxing patella, the tape is applied with the knee in a relaxed extended position on the plinth. The tape for these individuals, which is always positioned on the superior half of the patella, consists of a tilt and glide tape, followed by an internal rotation of the inferior pole of patella (care must be taken not to push the inferior pole of the patella into the fat pad when applying this tape) and external rotation of the superior pole. The last 2 pieces of tape are to decrease the contraction of the vastus lateralis. This is done by anchoring the tape halfway around the back of the distal end of the femur, just above the patella and firmly squashing the distal end of the vastus lateralis and iliotibial band by creating a divot. Another piece can be placed just above the first piece. When trying to improve patellar tracking, it is imperative that there is more balance of the quadriceps muscle – strong muscles get stronger and weak stay weak. Thus, decreasing lateralis activity slightly will encourage VMO activity and improve quadriceps balance and the seating of the patella in the trochlea ^(15,16)^.

**Potential complications or adverse events**

The minor downside to taping is that it can cause skin issues. There are two different types of skin issues. The first, which is the most common, is a friction rub which is less likely with the acute dislocation of the patella, as these patients are not using their knees through a wide range of motion and are not regularly removing the tape. The friction rub is a skin breakdown (blister) on the medial aspect of the knee, mostly due to too vigorous application of the tape when the knee moves through full range and pulls against the tape, hence pulling on the skin, or due to too rapid removal of the tape. The solution is when removing the tape to peel the tape off slowly from superior to inferior (i.e. towards the toes) and to use the other hand to ease the tension off the skin. A protective spray or wipe can also be used before applying the tape, but this must be dry before the tape is applied, otherwise the tape will not stick.

The second skin issue is an allergic reaction, where the whole area becomes inflamed, raised and itchy. This is uncommon - about 5-10% of individuals have this problem. It is more common in individuals who have a history of allergy – eczema, asthma. There is often a three-week delay before the irritation begins, because if the patient hasn’t been exposed to the allergen before (it’s usually the zinc oxide adhesive of the rigid tape), then the reaction is initially B cell, not T cell mediated. If the clinician suspects that this may be an issue they can use a skin preparation first, followed by 2 layers of the hypoallergenic tape (it’s the weave of that tape which can allow the zinc oxide glue of the rigid tape to seep through) and then apply the rigid tape. If the skin is becoming itchy an oral, over the counter, antihistamine tablet will usually settle the itch and stop the allergic response.

**Return-to-sport guidelines/criteria**

Taping for six weeks as described above after acute patellar dislocation allows early functional rehabilitation. The tape is easy to apply, is inexpensive and gives the patient a great deal of confidence which minimises fear of pain, so will enhance VMO activity ^(7)^.

With rehabilitation, patients are aiming to improve the synergistic firing of their muscles so the right muscles fire at the right time, particularly the VMO to improve the seating of the patella into the trochlea. Patients need to subtly change the motor program before they attempt to commence strengthening programs, as strong muscles get stronger and weak stay weak. When the patient is six weeks post-acute dislocation, they can commence performing small range of motion knee bends slowly in inner range to 30^o^ while controlling the return to extension, so they concentrate on soft knees not locked knees ^(8,9)^. When performing these movements, the patient needs to concentrate on their limb alignment ^(8.9)^. This is because weight bearing or closed kinetic chain (CKC) training is more effective than open chain exercises (OKC), as it promotes a more simultaneous onset of EMG activity of the quadriceps compared with OKC ^(17)^. In OKC, RF activates earliest, while the VMO is activated last with smaller amplitude than in CKC, so CKC exercise promotes a more balanced quadriceps activation and increases the thickness of the VMO, than OKC exercises ^(9,18)^. Additionally, CKC training allows simultaneous training not only of the vasti, but also the gluteals and trunk muscles to control the limb position in weight bearing ^(17)^.

After 3 months patients may commence more controlled loaded limb strengthening activities, provided they are feeling more confident about their knee stability. At four – five months they can commence, depending on their stability and confidence, straight line running and at six months they can start change of direction movements with a gradual introduction into their sporting activities. At nine – twelve months, they should be ready to return to their previous sporting activities, but they must be taped, at all times before they train or play (Figure 1). The tape needs to be applied about twenty minutes before playing sport so that it has time to set properly.

**Outcomes of taping**

Rood et al ^(7)^ followed 18 patients for 5 years after their patients were randomized to either immediate taping for the first 6 weeks or the usual initial non-operative intervention of a cylinder cast in the first 6 weeks. Each group had the same rehabilitation protocol. Taping resulted in a significantly better Lysholm score at the six- and twelve-week follow-up (p = 0.001). The difference remained at the five-year follow-up (p = 0.008). There were no cases of re-dislocation in the taping group.

**References**

1. Baryeh K, Getachew F. [Patella dislocation: an overview.](https://pubmed.ncbi.nlm.nih.gov/34431342/)Br J Hosp Med 2021 Aug 2;82(8):1-10. doi: 10.12968
2. Duthon VB. [Acute traumatic patellar dislocation.](https://pubmed.ncbi.nlm.nih.gov/25592052/) Orthop Traumatol Surg Res. 2015 Feb;101(1 Suppl):S59-67. doi: 10.1016
3. Sanders TL, Pareek A, Hewett TE, Stuart MJ, Dahm DL, Krych AJ. [Incidence of First-Time Lateral Patellar Dislocation: A 21-Year Population-Based Study.](https://pubmed.ncbi.nlm.nih.gov/28795924/) Sports Health. 2018 Mar/Apr;10(2):146-151. doi: 10.1177
4. Smith TO, Gaukroger A, Metcalfe A, Hing CB. [Surgical versus non-surgical interventions for treating patellar dislocation.](https://pubmed.ncbi.nlm.nih.gov/36692346/)Cochrane Database Syst Rev. 2023 Jan 24;1(1):CD008106
5. Tedeschi R, Platano D, Giorgi F, Donati D.J [To Operate or Not? Evaluating the Best Approach for First-Time Patellar Dislocations: A Review.](https://pubmed.ncbi.nlm.nih.gov/39336921/) Clin Med. 2024 Sep 13;13(18):5434
6. Fuller JA, Hammil HL, Pronschinske KJ, Durall CJ. [Operative Versus Nonoperative Treatment After Acute Patellar Dislocation: Which Is More Effective at Reducing Recurrence in Adolescents?](https://pubmed.ncbi.nlm.nih.gov/28872404/) J Sport Rehabil. 2018 Nov 1;27(6):601-604.
7. Rood A, Boons H, Ploegmakers J, van der Stappen W, Koëter S. Tape versus cast for non-operative treatment of primary patellar dislocation: a randomized controlled trial. Arch Orthop Trauma Surg 2012;132:1199–1203.
8. McConnell J. Rehabilitation and nonoperative treatment of patellar instability. Sports Med Arthrosc. 2007 15(2):95-104.
9. McConnell J. 2022. Management of anterior knee pain from the physical therapist´s perspective, chapter 8 in Anterior Knee pain and Patellar Instability ed V Sanchis-Alfonso, Springer
10. McCulloch PC, Bott A, Ramkumar PN, et al. 2014. Strain within the native and reconstructed MPFL during knee flexion. J Knee Surg.;27(2):125-31
11. Dragoo JL, Johnson C, McConnell J. [Evaluation and treatment of disorders of the infrapatellar fat pad.](https://pubmed.ncbi.nlm.nih.gov/22149697/) Sports Med. 2012 Jan 1;42(1):51-67. doi: 10.2165
12. Leese J, Davies DC. [An investigation of the anatomy of the infrapatellar fat pad and its possible involvement in anterior pain syndrome: a cadaveric study.](https://pubmed.ncbi.nlm.nih.gov/32159227/) J Anat. 2020 Jul;237(1):20-28. doi: 10.111
13. Stephen JM, Sopher R, Tullie S, Amis AA, Ball S, Williams A. [The infrapatellar fat pad is a dynamic and mobile structure, which deforms during knee motion, and has proximal extensions which wrap around the patella.](https://pubmed.ncbi.nlm.nih.gov/29679117/)Knee Surg Sports Traumatol Arthrosc. 2018 Nov;26(11):3515-3524. doi: 10.1007
14. Derasari A, Brindle TJ, Alter KE, Sheehan FT McConnell taping shifts the patella inferiorly in patients with patellofemoral pain: a dynamic magnetic resonance imaging study. Phys Ther. 2010;90(3):411-9
15. Tobin S, Robinson G. The effect of McConnell’s vastus lateralis inhibition taping technique on vastus lateralis and vastus medialis obliquus activity. Physiotherapy 2000, 26(4):173–183
16. McCarthy Persson U, Fleming HF, Caulfield B. The effect of vastus lateralis tape on muscle activity during stair climbing. Man Ther. 2009 Jun;14(3):330-7. doi: 10.1016
17. [Stensdotter AK, Hodges PW, Mellor R, Sundelin G, Hager-Ross C. 2003.](http://www.ncbi.nlm.nih.gov/entrez/query.fcgi?db=pubmed&cmd=Retrieve&dopt=AbstractPlus&list_uids=14652500&query_hl=34&itool=pubmed_docsum) Quadriceps activation in closed and in open kinetic chain exercise. Med Sci Sports Exerc 35(12):2043-7.
18. Cheon S., Lee J.-H., Jun H.-P., An Y. W., Chang E. Acute Effects of Open Kinetic Chain Exercise versus Those of Closed Kinetic Chain Exercise on Quadriceps Muscle Thickness in Healthy Adults. Int J Environ Res Public Health. 2020 Jun 29;17(13):4669
